# Allergic sensitization among Danish infants at 13 months of age

**DOI:** 10.1002/iid3.260

**Published:** 2019-06-12

**Authors:** Lisbeth M. Thøstesen, Poul‐Erik Kofoed

**Affiliations:** ^1^ Department of Pediatrics Herning Hospital Herning Denmark; ^2^ CVIVA, Research Center for Vitamins and Vaccines Statens Serum Institut København Denmark; ^3^ OPEN, Institute of Clinical Research University of Southern Denmark/Odense University Hospital Odense Denmark; ^4^ Department of Pediatrics Kolding Hospital Kolding Denmark; ^5^ Institute of Regional Health Research University of Southern Denmark Odense Denmark

**Keywords:** atopic dermatitis, infants, sensitization

## Abstract

**Background:**

Sensitization means elevated number of specific immunoglobulin E, either measured by skin prick test or in blood samples. Sensitization is associated with, but not synonymous with, allergic disease.

**Methods:**

The Danish Calmette Study was conducted from 2012 to 2015 at three Danish hospitals, with the aim of exploring nonspecific effects of neonatal Bacillus Calmette‐Guérin vaccination. Participants at Kolding Hospital were invited to have a blood sample analyzed for allergic sensitization at 13 months of age. Telephone interviews gave information about allergic symptoms, and the children were examined for signs of atopic dermatitis at 3 and 13 months.

**Results:**

Of the 1241 children included in the Danish Calmette Study in Kolding 1066 (86%) had a blood sample drawn, representing 36% of the invited families. The blood sample cohort had a relatively high percentage of atopic predisposition (66.6%) and most mothers had a medium or long education. We found 90 infants (8.4%) to be sensitized, with sensitization against food items (milk, egg, peanut, and hazelnut) being the most common. Atopic dermatitis was found in 19% of the children, and it was significantly associated with sensitization against egg, peanut, wheat, cat, and dog.

**Conclusion:**

In a partly selected Danish cohort, sensitization was present in 8% at 13 months of age, especially sensitization against food items. Children with atopic dermatitis were significantly more sensitized (16.6%). However, most sensitized children did not have any allergic symptoms at this age.

**Abbreviations**
BCG: Bacillus Calmette‐Guérin
IgE: immunoglobulin E

## 1 INTRODUCTION

Sensitized individuals have an elevated number of specific immunoglobulin E (IgE), either measured by skin prick test or in blood samples. Although sensitization is not synonymous with allergic disease,^1^ the frequency of sensitization in the background population could be a proxy for the possible impact of allergic disease.

Only few studies have studied sensitization in blood samples from unselected cohorts without any suspicion of allergic diseases examining blood samples. In a Danish study from 2013 an unselected cohort of 276 newborns was revisited at 26 years of age,^2^ providing rates of sensitization and diagnoses of atopic diseases at different ages. At 1.5 years of age, 89% had a blood sample drawn, demonstrating a sensitization rate of 8%.^2^

Among children with allergy‐like symptoms, the sensitization rate is much higher: a study from 2015 in Italy and Spain found half of the children sensitized.^3^ Among the included children below 5 years of age the sensitization rate was 47% in Italy and 18% in Spain.^3^

In the frame of a big randomized clinical trial, the sensitization rate was measured in a cohort of partly unselected children at 13 months of age.

## 2 METHODS

The Danish Calmette‐Study was a multicenter randomized clinical trial conducted 2012‐2015 at three Danish hospitals, exploring the nonspecific effects of neonatal vaccination with Bacillus Calmette‐Guérin (BCG).^4^ Inclusion criteria were: gestational age at least 32 weeks, birth weight at least 1000 g, and a parentally signed consent form. Pregnant women planned to give birth from 15th October 2012 to 17th November 2013 were invited to participate.^4^ Kolding Hospital is a regional hospital, taking care of most pregnancy‐related complications, though selected pregnant women were referred to give birth at a university clinic.

The included families were followed for 13 months with two telephone interviews and two clinical examinations, both at 3 and 13 months of age.^4^ Participants followed in Kolding were invited to have a blood sample analyzed for allergic sensitization at 13 months of age. The blood samples were drawn by laboratory staff and analyzed at Thermo Fischer Scientific.^5^ In case of a positive screen test (Phadiatop infant ≥0.20 kU/L), the blood samples were analyzed for specific IgE antibodies against food allergens (milk, egg, peanut, wheat, and hazelnut) and inhalant allergens (house dust mites, dog, cat, birch, and grass). Sensitization was defined as specific IgE antibodies of ≥0.35 kU/L.

Included families whose children had no sensitization were informed by a letter. Parents to children with sensitization were contacted by phone, and in case of possible allergic symptoms consistent with the sensitization pattern they were referred to the Pediatric Allergy Clinic in Kolding.

In the two follow‐up telephone interviews the parents were asked if a doctor had ever given the child a diagnosis of atopic dermatitis, and at 3 and 13 months of age a clinical examination was performed for signs of atopic dermatitis.^4^, ^6^

In the Danish Calmette Study, we found no effect of neonatal BCG vaccination on allergic sensitization or on reported food allergy.^5^ Therefore, in the present descriptive paper we have pooled all the children included in the blood sample cohort in Kolding, regardless of their randomization group.

### 2.1 Statistical analysis

Tabulation with *χ* ^2^ tests were used for testing statistical significant differences, defined as *P* < .05 using STATA 14 (StataCorp, College Station, TX).

### 2.2 Ethics statement

The Danish Calmette Study, including the blood sample study in Kolding, was approved by the Danish Data Protection Board (2009‐41‐4141), the Committee on Biomedical Research Ethics (H‐3‐2010‐087), and the Danish Medicines Agency (2612‐4356. EudraCT 2010‐021979‐85. Protocol 2009‐323). The study was registered at www.ClinicalTrials.gov (NCT01694108) and supervised by the Good Clinical Practice Units and by an independent Data and Safety Monitoring Board. All parents gave verbal and written consent for participation in the study, and separate consent for the blood sampling.

## 3 RESULTS

**Figure 1:**
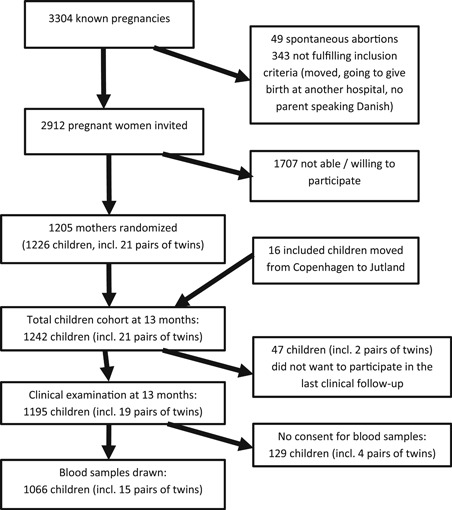
Flowchart of the blood sample cohort

The flow chart of the Kolding blood sample cohort is shown in Figure 1. Of the 2912 invited pregnant women, 1204 were randomized in the Danish Calmette Study (42.6%), of these 21 gave birth to twins. During follow up 16 children included in Copenhagen moved to Jutland and were thus followed in Kolding for the final clinical examinations, including the possibility of having the mentioned blood sample drawn.

We had a high follow‐up rate in our study, as a total of 1195 of 1242 included children (96%) were seen for the clinical examination at 13 months of age, including 19 pairs of twins. Of the 1195 children seen 1066 (86%) had a blood sample drawn. A total of 1052 (36.1%) of the 2912 invited families accepted to participate in the blood sample substudy, together with 14 families moving from other inclusion sites (Figure 1).

One of the aims of the Danish Calmette Study was to examine a possible protective effect against atopic dermatitis,^6^ recurrent wheeze,^7^ and food allergy.^5^ Families with atopic diseases were, therefore, more prone to participate in the study, as were families with well‐educated mothers. Table 1 shows the background characteristics of the 1066 children in the present substudy.

**Table 1:** Background characteristics for the blood sample cohort

	No.	%
	1066	100
Mother's age, y, median (10‐90 percentiles)	31	25‐37
*Mother's highest education*		
No higher education	358	33.6
Basic school	59	5.5
High school	83	7.8
Nontheoretical schooling	216	20.3
Short/medium higher education	556	52.1
Short theoretical schooling	92	8.6
Bachelor level	464	43.5
Long higher education (master level or PhD)	152	14.3
Parents living together [1]	1020	95.8
Other ethnicity than Danish^a^, [4]	96	9.0
Atopic predisposition [15]	700	66.6
Maternal atopic disease [2]	422	39.7
Paternal atopic disease [27]	349	33.6
Parental atopic disease [17]	638	60.8
Siblings with atopic disease	183	17.2
Fur animals in the household	529	49.6
Siblings	571	53.6
Cesarean section	219	20.5
Gestational age, d, median (10‐90 percentiles)	282	267‐290
Premature (gestational age <37 wk)	39	3.7
Sex: boy	544	51.0
Multiple birth (in percentage of 1051 pregnancies)	15	1.4
Birth weight, g, median (10‐90 percentiles)	3560	2900‐4180
Fully breastfed at 3 mo [2]	607	57.0
Use of hydrolyzed infant formulas [4]	48	4.5
Age in months at first introduction to food (other than milk); median (10‐90 percentiles)	4.0	4.0‐6.0
Started daycare before 13 mo	997	93.5
Parent(s) ever smoked since birth of child [6]	270	25.5
Parent(s) smoking daily; or indoor smoking [5]	199	18.8
Mother smoking daily [7]	92	8.7
Father smoking daily [8]	156	14.7
Indoor smoking	25	2.4
Clinically diagnosed atopic dermatitis	205	19.2
Age in months at blood sampling (10‐90 percentiles)	12.9	12.4‐13.6

*Note*: Missing values are provided in square brackets.

^a^ At least one grandparent originating from another country than Denmark

### 3.1 Sensitization

A total of 143 children had a positive screen test, that is, Phadiatop infant ≥0.20 kU/L. Ninety of these children (8.4% of the blood sample cohort) had at least one specific IgE against the mentioned food or inhalant allergens ≥0.35 kU/L. Sensitization against food allergens was far more common than sensitization against inhalant allergens (Table 2).

**Table 2:** Sensitization at 13 months of age

	No	% of whole cohort (1066 children)	% of sensitized children (90 children)	Specific IgE: mean (range)	Specific IgE: median (10‐90 percentiles)
Sensitization against food allergens	80	7.5	88.9		
Milk	58	5.4	64.4	1.40 (0.35‐9.31)	0.70 (0.39‐3.25)
Egg	25	2.3	27.8	5.17 (0.36‐88.16)	0.98 (0.42‐4.53)
Peanut	15	1.4	16.7	2.58 (0.4‐11.53)	0.7 (0.43‐8.05)
Hazelnut	13	1.2	14.4	4.49 (0.36‐24.99)	0.76 (0.41‐15.25)
Wheat	7	0.7	7.8	2.79 (0.5‐10.99)	1.05 (0.5‐10.99)
Sensitization against inhalant allergens	14	1.3	17.7		
House dust mites	6	0.6	6.7	1.44 (0.37‐3.27)	0.64 (0.37‐3.27)
Dog	6	0.6	6.7	19.16 (0.57‐70.06)	2.33 (0.57‐70.06)
Cat	5	0.5	5.6	1.40 (0.35‐3.7)	0.66 (0.35‐3.7)
Grass^a^	2	0.2	2.2	0.39 (0.38‐0.39)	0.39 (0.38‐0.39)
Birch^a^	0	…	…	…	…
Sensitization against food and inhalant allergens	7	0.7	7.8		

^a^ One child was not analyzed for sensitization against grass and birch due to a too small blood sample

### 3.2 Effect modifiers

As shown in Table 3 we found no effect of the predefined effect modifiers on the frequency of sensitization. Not even atopic predisposition increased the rate of sensitization. In our study boys (54 of 544 = 9.9%) were more prone to be sensitized than girls (36 of 522 = 6.9%), but the difference was not statistically significant (*P* = .075).

**Table 3:** Possible effect modifiers in relation to sensitization

	Sensitized/total number (%)	*P* value (*χ* ^2^)
Whole cohort	90/1066 (8.4)	
Sex		.075
Male	54/490 (9.9)	
Female	36/486 (6.9)	
Gestational age (GA) at birth		.179
Prematurity (GA 32 + 0 to 36 + 6)	1/39 (2.6)	
Mature (GA at least 37 + 0)	89/938 (8.7)	
Atopic predisposition [15]		.869
With atopic predisposition	58/709 (8.2)	
Without atopic predisposition	29/342 (8.5)	
Maternal BCG vaccination [12]		.374
Mother BCG vaccinated	13/122 (10.7)	
Mother not BCG vaccinated	77/932 (8.3)	
Ethnicity [4]		.116
Danish ethnicity	12/96 (12.5)	
Non‐Danish ethnicity	76/966 (7.9)	
Siblings		.692
No older siblings	50/571 (8.8)	
At least one older sibling	40/495 (8.1)	
Pets at home		.557
Yes	42/529 (7.9)	
No	48/537 (8.9)	
Breastfeeding [2]		.554
Fully breastfed at 3 mo	54/607 (8.9)	
Not fully breastfed at 3 mo	36/457 (7.9)	
Use of hydrolyzed infant formula at 3 mo [4]		.971
Yes	4/48 (8.3)	
No	86/1014 (8.5)	
Smoking status by 13 mo of age [6]		.437
Ever smoked since birth of child	26/270 (9.6)	
Never smoked since birth of child	64/790 (8.1)	
Started daycare before 13 mo		.599
Yes	83/997 (8.3)	
No	7/69 (10.1)	

*Note*: Missing values are provided in square brackets

### 3.3 Association with atopic dermatitis

**Figure 2:**
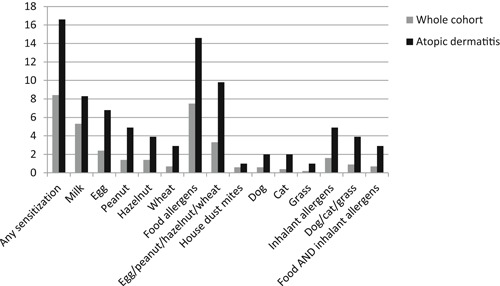
Percentage of sensitized children in the whole cohort respectively among children diagnosed with atopic dermatitis

Among the 1066 infants in our blood sample cohort, 205 (19.2%) were diagnosed with signs of atopic dermatitis, either by their own practitioner or at the clinical examinations in the Danish Calmette Study. As shown in Table 4, we found atopic dermatitis significantly associated with sensitization against egg, peanut, wheat, and cat. For all 10 allergens, children with atopic dermatitis were more sensitized than children without atopic dermatitis (Figure 2).

**Table 4:** Association between sensitization and atopic dermatitis

	All children (%)	Children without AD (%)	Children with AD (%)	*P* value (*χ* ^2^)
Any sensitization	90/1066 (8.4)	56/861 (6.5)	34/205 (16.6)	<.001
Milk	56/1066 (5.3)	39/861 (4.5)	17/205 (8.3)	.030
Egg	25/1066 (2.4)	11/861 (1.3)	14/205 (6.8)	<.001
Peanut	15/1066 (1.4)	5/861 (0.6)	10/205 (4.9)	<.001
Hazelnut	13/1066 (14)	5/861 (0.6)	8/205 (3.9)	<.001
Wheat	7/1066 (0.7)	1/861 (0.1)	6/205 (2.9)	<.001
Food allergens	80/1066 (7.5)	50/861 (5.8)	30/205 (14.6)	<.001
Egg/peanut/hazelnut/wheat	35/1066 (3.3)	15/861 (1.7)	20/205 (9.8)	<.001
House dust mites	6/1066 (0.6)	4/861 (0.5)	2/205 (1.0)	.379
Dog	6/1066 (0.6)	2/861 (0.2)	4/205 (2.0)	.003
Cat	4/1066 (0.4)	0	4/205 (2.0)	<.001
Grass	2/1065 (0.2)	0	2/205 (1.0)	.004
Inhalant allergens	17/1065 (1.6)	7/860 (0.8)	10/205 (4.9)	<.001
Dog/cat/grass	10/1065 (0.9)	2/860 (0.2)	8/205 (3.9)	<.001
Food and inhalant allergens	7/1066 (0.7)	1/861 (0.1)	6/205 (2.9)	<.001

Among children with atopic dermatitis 16.6% (34 of 205) were sensitized, compared with 6.5% (56 of 861) of children without atopic dermatitis. And among the sensitized children 37.8% (34 of 90) had atopic dermatitis, compared with 17.5% (171 of 976) of the not sensitized children. We thus found atopic dermatitis and sensitization highly associated, *P* < .001.

#### 3.3.1 Recurrent wheeze and house dust mites

Overall there was no association between sensitization and recurrent wheeze (*P* = .66). At this age, sensitization against house dust mites was neither correlated with atopic dermatitis (*P* = .38), nor with recurrent wheeze (*P* = .18). However, we only found six children sensitized against house dust mites.

### 3.4 Possible allergy

Of the 90 sensitized children, 21 (23.3%) were referred to the Pediatric Allergy Clinic because of possible allergic symptoms. Further, four children (4.5%) had previously had allergic symptoms, which had now waned. The majority of sensitized children (72.2%) had not had any allergic symptoms. Especially sensitization against milk was often seen without concomitant milk allergy. Thus, out of the 1066 children in the blood sample cohort, only 21 (2.0%) had both sensitization and relevant possible allergic symptoms with a need of referral to the Pediatric Allergy Clinic.

### 3.5 Case reports from the pediatric allergy clinic

A boy with an impressively high specific IgE (Phadiatop infant) of 72.9 kU/L was referred to the Pediatric Allergy Clinic, but he had no allergic symptoms and was thus only sensitized.

A girl with specific IgE against egg just above the cut‐off level (0.36 kU/L) showed up to be clearly allergic to egg with a big reaction on the skin prick test and a clearly positive egg challenge.

A boy with sensitization against peanut showed out to be allergic with a low threshold. Once he experienced prompt allergic reaction to accidental touching of his skin with a minimal amount of peanut butter. His parents were grateful for knowing what was going on and thus being able to react relevantly.

## 4 DISCUSSION

Sensitization is not synonymous with allergy, as allergy requires allergic symptoms.^1^ The most common sensitization found in our study was against milk, but most of these children drank cow's milk without having any allergic symptoms.

In a recent study from Moscow 16% of males and 25% of females aged 0 to 1 year were sensitized; however the Russian study was performed among children referred with allergic symptoms.^8^ In our partly unselected cohort we found a sensitization rate of 8%, and in contrast with the study from Moscow we found more sensitization among boys (9.9%) than among girls (6.9%).

Our sensitization rate of 8% at 13 months of age corresponds perfectly with the 8% sensitized children at 1.5 years of age in the Danish study described by Nissen et al.^2^ They included 276 newborns in 1985, and we draw blood samples from our cohort of 1066 children in 2014, almost 30 years later. Although we had 67% with an atopic predisposition, compared to 35% in the study of Nissen et al, we found the same sensitization rate at 13 months of age as they found at 18 months. We found 19% with atopic dermatitis, compared to 13% in the study of Nissen et al.^2^ The reason for more children with atopic dermatitis in our study might be the greater proportion of children with atopic predisposition. So there has been no major change in the prevalence of neither sensitization nor atopic dermatitis during the past 30 years.

Our cohort was not entirely unselected, as families with atopic diseases were more prone to participate. The reason for this self‐selection into the study was primarily the hypothesis, that neonatal BCG vaccination might reduce the risk of atopic diseases later in life. As reported elsewhere we found no such general protection of the vaccination.^5^, ^6^, ^7^ As the BCG vaccination was not associated with the atopic outcomes, we have pooled all children in the present article, regardless of their randomization group.

We found a significant association between atopic dermatitis and sensitization. A Swedish study from 2016 found sensitization associated with childhood eczema, and after the age of 4 years also with rhinitis and asthma. However, 23% of the sensitized children never developed atopic diseases during childhood.^9^

A recently published Australian study performed skin prick tests in 706 children at 1, 3, and 6 years of age.^10^ All the included children had a mother, father or sibling with an allergic disease (self‐reported history of medically diagnosed eczema, asthma, or hay fever). At 1 year of age 17% of the children were sensitized against at least one food allergen or one inhalant allergen.^10^ The Australian children had similar allergic predisposition as the 709 children in our study with atopic predisposition (Table 3), however only 8.2% of our children were sensitized.

A generally accepted cut‐off value for Phadiatop infant is 0.35 kU/L. We chose 0.20 kU/L as our screening test to include children with a Phadiatop infant below the normal cut‐off value of 0.35 kU/L, but with a single specific IgE against a specific allergen above this limit. In a recently published Swedish study there was an association between low sensitization at 6 months of age (especially to egg) and sensitization to aeroallergens at 5 years of age.^11^ Our cohort was only followed for 13 months, but some children might develop other sensitizations later in life.

Although specific IgE measurement in a blood sample and the results of a skin prick test are often considered equivalent, skin prick tests might be more sensitive. In an American study from 2008 performed among adult laboratory workers, Sharma et al^12^ found skin prick test more useful than specific IgE measurement in the diagnosis of mouse allergy.

In a cohort of (adult) patients with allergic rhinitis Ciprandi et al^13^ concluded that specific IgE seemed more appropriate than skin prick test in multisensitized patients.

At 13 months of age sensitization was not significantly associated with atopic predisposition. This could be due to the relatively young age of the children in our study. In the Danish cohort from Odense followed for 26 years, food allergy and eczema were the most prevalent allergic diseases in early childhood, with an increasing number of study participants being affected by asthma or rhinoconjunctivitis later on.^2^

In our study, sensitization associated with possible allergy was primarily found among egg and peanut sensitized infants. Sensitization against egg or peanut thus had a greater clinical importance and was more often associated with atopic dermatitis than sensitization against milk.

With only 2% of our blood sampling cohort needing referral to the Pediatric Allergy Clinic we recommend specific IgE to be measured only when there are possible allergic symptoms. Atopic dermatitis can be a symptom of food allergy.

### 4.1 Strengths

We had impressively high follow‐up numbers in our study, with 86% of the included children having a blood sample drawn. The 1066 children included in the present blood sample study represent 34% of the birth cohort during the inclusion period.

### 4.2 Weaknesses

Our cohort had more atopic predisposition and a higher maternal education level than the general background population, and thus our results might not be directly transferable to the background population.

We only followed the children for 13 months and thus we do not have any data on a possible correlation with later asthma or other atopic diseases.

## 5 CONCLUSION

In a partly selected Danish cohort sensitization (especially against food items) was present in 8% at 13 months of age. Of the sensitized children 23.3% had allergic symptoms. However, most sensitized children did not have any allergic symptoms at this age.

## CONFLICT OF INTERESTS

The authors declare that there are no conflict of interests.

## DATA ACCESSIBILITY

The data set generated and analyzed during the current study are not publicly available but are available from the corresponding author upon reasonable request.
